# Cold induced expression of a novel levansucrase gene *sacB1* enhances exopolysaccharide production and stress resilience in *Leuconostoc mesenteroides*

**DOI:** 10.1038/s41598-025-04141-x

**Published:** 2025-07-02

**Authors:** Miguel Fernandez de Ullivarri, Colin Buttimer, Janneke Wijman, Eelco Heintz, Paul Ross, Matthew P. McCusker, Colin Hill

**Affiliations:** 1 APC Microbiome Ireland, School of Microbiology, University College Cork, Cork, T12 YT20 Ireland; 2 Niacet, A Kerry® Company, 4000 AB Tiel, The Netherlands; 3 Kerry Taste & Nutrition, Global Technology & Innovation Centre, Millennium Business Park, Naas, Co. Kildare W91 W923 Ireland

**Keywords:** *Leuconostoc mesenteroides*, Exopolysaccharide, Levansucrase, Cold stress, Sucrose-induced metabolism, Applied microbiology, Bacterial physiology, Biofilms, Environmental microbiology, Food microbiology, Bacterial genes, Industrial microbiology, Biopolymers

## Abstract

Exopolysaccharides (EPS) play critical roles in microbial survival, stress adaptation, and biofilm formation across diverse environments. In food-associated bacteria such as *Leuconostoc mesenteroides*, understanding the regulation of EPS production under environmental stress is important for both spoilage control and industrial applications. However, the mechanisms linking cold stress to EPS biosynthesis remain poorly understood. Here, we show that sucrose and low temperature (8 °C) trigger a metabolic shift from dextran-only to combined dextran and levan biosynthesis in four meat-borne *Leuc. mesenteroides* strains. Two high EPS-producing strains (HEPRs) possess the *sacB_1* gene, which encodes a previously uncharacterized levansucrase absent from low EPS-producing strains (LEPRs) that only carry the *levS* gene. This is the first study to describe the role of *sacB_1* in cold-induced EPS production. Notably, *sacB_1* was also identified in *Leuc. mesenteroides* strains isolated from plant-based fermentations such as kimchi and birch sap, but the HEPR strains analyzed here are the only known meat-derived isolates to carry this gene. Genomic analyses revealed highly conserved biosynthetic clusters for dextran, heteropolysaccharide, and levan. Gene expression profiling showed that *levS* and *sacB_1* were upregulated at 8 °C, while *dsrD* expression was favoured at 25 °C. Cold-induced sucrose metabolism, characterized by high expression of *levS*, *sacB_1*, and *dsrD*, enhanced cell viability under oxidative stress. Furthermore, heterologous expression of *sacB_1* in *Leuc. mesenteroides* and *Lactococcus lactis* improved resilience under cold and high-aeration conditions, confirming the protective role of levan. These findings advance the understanding of temperature-dependent EPS regulation in LAB and highlight sucrase diversity as a key factor in microbial adaptation to environmental stress.

**Supplementary Information:**

The online version contains supplementary material available at 10.1038/s41598-025-04141-x.

## Introduction

Microbial exopolysaccharides (EPS) are carbohydrate polymers secreted by bacteria as either capsular polysaccharides (CPS), which remain attached to the cell surface, or released EPS (rEPS), which disperse into the surrounding medium. EPS contribute to bacterial physiology by providing structural protection and mediating interactions with the environment. CPS shield cells from environmental stress, immune responses, or desiccation^1^while rEPS are integral to biofilm matrices, supporting cell–cell adhesion and collective resilience^2^. EPS are classified by composition into homopolysaccharides (HoPS), composed of a single monosaccharide such as dextran or levan, and heteropolysaccharides (HePS), which consist of complex repeating units of multiple sugars (e.g., alginate, CPS)^3^.

*Leuconostoc mesenteroides* is a lactic acid bacterium (LAB) known for producing rEPS with industrial applications^4^. Many *Leuconostoc* strains synthesize HoPS—such as dextran, mutan, inulin, and alternan^5^—using sucrase-type enzymes that convert sucrose into glucans or fructans via transglycosylation^6^. These enzymes, classified as glucansucrases (GH70) or fructansucrases (GH68), transfer a sugar residue from sucrose to a growing HoPS chain, releasing the remaining monosaccharide^6^. Excess HoPS production in sucrose- or raffinose-containing media leads to visibly slimy colonies on agar surfaces^7^.

HoPS are synthesized extracellularly by sucrases, either soluble or cell-wall-bound^8^. In contrast, HePS are synthesized intracellularly via the Wzy-dependent pathway, which involves the formation of repeating units, membrane translocation, and polymerization outside the cell^3,9^. The HoPS biosynthetic pathway has been described in several LAB genera including *Leuconostoc*, *Lactobacillus*, *Pediococcus*, *Streptococcus*, and *Weissella*, while HePS biosynthesis via the Wzy-dependent pathway is mainly characterized in *Lactococcus*, *Lactobacillus*, and *Streptococcus*^10^. Recent findings also indicate the capacity of *Leuconostoc* strains to produce HePS via this route^9^.

Fructans such as levan and dextran are strongly associated with microbial stress tolerance, biofilm formation, and antioxidant properties. For example, levan stabilizes cell membranes under thermal and chemical stress in *Limosilactobacillus reuteri*^11,12^and supports tolerance to osmotic and gastrointestinal stress in *Bacillus subtilis*^13^. Levans also show antioxidant activity in species such as *B. subtilis*, *Acetobacter xylinum*, and *Leuconostoc*^14–18^. In biofilms, levans contribute to matrix architecture and nutrient retention, as observed in *B. subtilis*, *Erwinia amylovora*, and *Pseudomonas syringae*^19–22^. In *P. syringae*, for instance, levan accumulates in cell-depleted regions of the biofilm, aiding maturation and nutrient distribution^21^. Dextrans, similarly, are EPS components in *Leuconostoc* biofilms^23,24^ and possess antioxidant functions comparable to levan^18,25^.

In *Leuc. mesenteroides*, EPS production is influenced by environmental factors such as carbon source, pH, oxygen levels, and temperature^8,26–28^. While cold exposure has been linked to dextran production in some *Leuconostoc* strains, its broader role in EPS regulation and microbial survival remains poorly defined. Notably, no prior studies have addressed the effect of cold on fructan production in this genus. This question is particularly relevant in the meat industry, where excessive EPS leads to undesirable slime formation, affecting the appearance and texture of products^29^.

Understanding how *Leuc. mesenteroides* regulates EPS biosynthesis under cold stress is essential to elucidate microbial adaptation mechanisms and could have direct applications in controlling spoilage during refrigerated food storage. Furthermore, identifying novel sucrase genes involved in cold-induced EPS production could expand current knowledge of lactic acid bacteria physiology and sucrase diversity.

In this work, we investigated the genetic and environmental regulation of EPS production in *Leuc. mesenteroides* strains isolated from cold-stored meat sausages, with a focus on the role of levansucrases under cold stress.

## Materials and methods

### Culture media and strains

Man Rogosa Sharpe (MRS), M17 without lactose, and Luria-Bertani (LB) media, as well as glucose, sucrose, and fructose, were purchased from Merck (Germany) and used in liquid or solid form (1.5% agar).

MRS was chosen for initial isolation of slime producing strains as it promotes strong growth and visual EPS phenotype expression. In contrast, M17 was used in downstream experiments to reduce background EPS and better isolate the effects of sugar and temperature on EPS induction.

Four *Leuc. mesenteroides* strains (KS273, KS276, KS277 and KS279) called KS strains, were isolated from spoiled commercial cooked-meat sausages from individual packages from two different manufacturers bearing a slimy defect on their surface. Briefly, a sample from the slime on the sausages was collected with a sterile loop, streaked on MRS agar plates and incubated at 30 °C for 48 h. Individual colonies were sub-cultured under the same conditions to obtain pure cultures. Colonies were selected based on the presence of a slimy phenotype, assessed after 48 h of incubation. A sterile inoculation loop was gently touched to the colony surface and lifted vertically; colonies that produced a visible ropy thread ≥ 3 mm in length were considered positive for slimy phenotype and selected for further propagation. Other strains used in this study are listed in Table 1 and Supplementary Table S1.

**Table 1:** Bacterial strains used in this study

Species	Strain	Description	Reference/source
*Leuconostoc mesenteroides subsp. dextranicum*	KS273	Food borne; Low temp. strong slime former	This study
*Leuc. mesenteroides subsp. dextranicum*	KS276	Food borne; Low temp. weak slime former	This study
*Leuc. mesenteroides subsp. dextranicum*	KS277	Food borne; Low temp. weak slime former	This study
*Leuc. mesenteroides subsp. dextranicum*	KS279	Food borne; Low temp. strong slime former	This study
*Leuc. mesenteroides subsp. dextranicum*	KS276-SB1	KS276 carrying the plasmid pNZSB1:*sacB_1*	This study
*Leuc. mesenteroides subsp. dextranicum*	KS276-E	KS276 carrying the plasmid pNZSB1	This study
*Lactococcus lactis subsp. cremoris*	MG1363	Plasmid free *L. lactis* strain	Gasson, 1987^91^
*L. lactis subsp. cremoris*	MG1363-SB1	MG1363 carrying the plasmid pNZ44:*sacB_1*	This study
*L. lactis subsp. cremoris*	MG1363-E	MG1363 carrying the plasmid pNZ44	This study
*Escherichia coli*	TOP10	Plasmid free *E. coli* strain	Invitrogen

### Qualitative evaluation of the effect of carbon sources and temperature on EPS production

The *Leuc. mesenteroides* strains were cultured overnight at 30 °C in M17 broth supplemented with 0.1% glucose. Grown cultures were centrifuged at 4000×*g* at room temperature and cells were washed twice with 10 mL phosphate-buffered saline (PBS) and inoculated into microtiter plates to a final optical density (OD_600_) of 0.05. Each well contained 150 µL of M17 broth supplemented with either sucrose, glucose, or fructose at concentrations ranging from 0 to 5% in triplicate. The plates were sealed with plastic films and incubated at 8 °C, 25 °C, or 37 °C for 5 days. Colony morphology was then visually compared across the different conditions. To assess EPS production, a standardized ropy phenotype test was performed. After 5 days of incubation, a sterile 1-µL inoculation loop was carefully dipped into the surface of each culture and slowly lifted vertically. The formation of a continuous thread of extracellular material was visually assessed and measured against a millimetre ruler positioned adjacent to the loop. The maximum thread length before breaking was recorded. Each sample was tested in triplicate.

### Quantitative evaluation of the effect of carbon sources and temperature on EPS production

Response surface methodology (RSM) was applied to evaluate the combined effects of sugar type, sugar concentration, and incubation temperature on EPS production by *Leuc. mesenteroides* strains KS273 and KS276. A full factorial experimental design was implemented using Design Expert v11 software (Stat-Ease, USA) to quantify the individual and interactive effects of these variables. M17 broth was supplemented with 0–5% (w/glucose or sucrose, and 20 mL of medium was inoculated with each strain to a final optical density (OD₆₀₀) of 0.05. Cultures were incubated at 8 °C, 25 °C, or 37 °C for 5 days without shaking. Following fermentation, EPS was extracted and semi-purified as described previously by Yáñez-Fernández et al.^28^ with modifications. Fermented broth (20 mL) was heated at 100 °C for 15 min and centrifuged at 4000×*g* for 30 min at 4 °C. The supernatant was collected and treated with 10% (v/v) 12 M HCl for 5 min at 70 °C under continuous stirring. After a second centrifugation step (4000×*g*, 30 min), the resulting supernatant was mixed with two volumes of ice-cold absolute ethanol and incubated overnight at − 20 °C with mild agitation. The precipitated material was recovered by centrifugation at 4000×*g* for 30 min, and the pellet was resuspended in a minimal volume of Milli-Q water. This resuspension–precipitation cycle with ice-cold ethanol (2:1, v/v) was repeated once more, with overnight incubations at – 20 °C and centrifugation steps identical to those described above. After the final precipitation, the pellet was resuspended in Milli-Q water and dialysed against Milli-Q water for 3 days at 4 °C using 12–14 kDa molecular weight cut-off (MWCO) dialysis tubing (Sigma-Aldrich), with daily water changes. The dialysed EPS samples were subsequently oven-dried at 50 °C for water and ethanol removal, and the dry weight was then measured on a precision scale.

### Whole genome sequencing (WGS) and bioinformatic analysis

DNA from the four strains was extracted by growing cultures overnight in 10 mL of M17 broth supplemented with 0.5% glucose at 30 °C. Genomic DNA was extracted using a GenElute Bacterial Genomic DNA Kit (Merck, Rahway, JN, USA). Genomes were subjected to whole genome sequencing with the Illumina MiSeq Sequencing System (San Diego, CA, USA) performed by Novogene (Cambridge, UK). Raw reads were downloaded in FASTQ format, quality control was run on the reads using FastQC v0.11.9 and Fastp v0.23.2. The genomes were assembled using Genome Assembly tool from the Pathosystems Resource Integration Center (PATRIC, http://www.patricbrc.org) analysis platform and annotated using NCBI PGAP^30^. Assembled genomes were deposited in GenBank under BioSample accessions SAMN46921126, SAMN46921127, SAMN46921128, SAMN46921129. Nonredundant Protein Database (NR), Swiss-Prot, and carbohydrate-active enzymes^31^ databases were used to contrast and complement gene functions. Conserved domains of GH68 and GH70 genes were analysed using the NCBI Conserved Domain Database^32^ and InterPro (https://www.ebi.ac.uk/interpro/search/sequence/).

### Pangenomic analysis

A pan-genome analysis was conducted among the four strains using Roary v3.13.0 to identify genomic differences among the four strains^33^. GFF files from annotation were used as input and run with a 95% sequence identity cutoff for grouping genes into orthologous clusters and identifying core and accessory genes. The resulting pan-genome matrix was used to identify genomic differences between strains. Figures of the proposed HePS-levan and glucan biosynthetic gene clusters (HLBC and GBC, respectively) were created with Clinker tool in CAGECAT platform^34^. To compare HLBC and GBC across species within the *Leuconostoc* genus, the Cblaster tool from the CAGECAT platform was used to identify and visualize homologous gene clusters^35^. HLBC and GBC from the query strain KS273 were searched against a genomic database of *Leuconostoc* species, with a minimum identity threshold of 70%.

### RNA sequencing and bioinformatic analysis

*Leuc. mesenteroides* KS273 and KS276 were grown in falcon tubes in 15 mL M17 broth supplemented with either 1.5% glucose, 1.5% sucrose or without addition of sugar and grown at either 25–8 °C for 3 days. Cell pellets were harvested by centrifugation at 4000×*g* at the same temperature from which they were grown and immediately stored in 1 mL TRIzol reagent at – 80 °C to preserve RNA integrity. Total RNA extraction was performed with GeneJET RNA Purification Kit following the manufacturer’s protocol. RNA quality and concentration were assessed by Agilent 2100 Bioanalyzer, and only samples with RIN values > 8.0 were used for sequencing.

RNA-seq library preparation, sequencing, and preliminary data processing were performed by Azenta Life Sciences (Leipzig, Germany). rRNA depletion was carried out with Ribo-Zero^®^, stranded libraries were prepared and sequencing was performed using the Illumina NovaSeq 6000 platform in paired-end mode with a read length of 2 × 150 bp. Data quality was assessed through standard bioinformatics workflows. RNA-seq raw data were analysed using the DESeq2 package v1.38.3 in R v4.3.1 for differential gene expression analysis. First, raw sequencing reads were aligned to the sequenced and annotated genomes using HISAT2 v2.2.1, and counts were generated with featureCounts v2.0.1. The resulting count matrix was imported into DESeq2, where normalization and variance stabilization were performed. Differential expression was assessed by fitting a generalized linear model and applying Wald’s test for statistical significance. Genes with an adjusted *p*-value (Benjamini-Hochberg correction) of < 0.05 were considered differentially expressed.

### Construction of expression vectors for recombinant expression of *sacB_1*

All primers used for gene amplification are listed in Supplementary Table S2. PCR was performed with Phusion High-Fidelity DNA Polymerase (2× Master Mix, Thermo Scientific). Each 100 µL reaction included 50 µL of master mix, 0.5 µM primers, and 50 ng of template DNA. PCR protocol consisted of 35 cycles of 98 °C for 10 s, annealing at optimal temperature for 10 s, and 72 °C for 15–30 s per kb, with a final extension at 72 °C for 5 min. PCR products were analysed using agarose gel electrophoresis and purified with GeneJET PCR Purification Kit (Thermo Scientific).

Restriction-based cloning was performed using NcoI and XhoI (Thermo Scientific) and ligated with T4 DNA ligase (Thermo Scientific). Two *sacB_1* expression constructs were generated: one with constitutive expression under the native P44 promoter in the pNZ44 vector (MoBiTec GmbH), and a second cold-inducible version carrying the native promoter (*P_sacB_1*) inserted into a promoter-less pNZ44 backbone. The *sacB_1* gene and *P_sacB_1* fragment were amplified from genomic DNA of *Leuc. mesenteroides* KS273 using primers NcoI-Lev2-for/XhoI-Lev2-rev and NcoI-Prom_Lev2-for/XhoI-Lev2-rev, respectively. The promoter-less pNZ44 vector backbone was amplified using primers XhoI-pNZ-for/NcoI-pNZ-rev.

Ligation mixtures (3 µL) were transformed into *E. coli* TOP10 chemically competent cells (Invitrogen, Cat. No. C404010) using a standard heat-shock protocol. Following 1 h of recovery in 500 µL LB broth at 37 °C with shaking at 180 rpm, 100 µL of the culture was plated onto LB agar supplemented with 10 µg/mL chloramphenicol (Cm10) and incubated at 37 °C for 48 h. Positive transformants were screened by colony PCR using primers pNZ44-for/pNZ44-rev, and plasmids were propagated in LB-Cm10 medium. Plasmid DNA was extracted using the QIAprep Spin Miniprep Kit (Qiagen, Cat. No. 27106) and fully sequenced at Plasmidsaurus (USA) to confirm sequence integrity.

Electrocompetent *Leuc. mesenteroides* KS276 and *Lactococcus lactis* MG1363 cells were prepared following the protocols of Seon-Ju et al. (2006)^36^ and Holo & Nes (1989)^37^respectively. Electroporation was performed using 2.5 kV and 25 µF in 2 mm gap cuvettes, introducing 1 µg of pNZ44:*sacB_1* or pNZSB1:*sacB_1* into 50 µL of competent cells. Following a 2 h recovery at 30 °C in M17 broth supplemented with 0.5% glucose (GM17), 100 µL of culture was spread onto GM17 agar plates containing 5 µg/mL chloramphenicol (Cm5). Prior testing confirmed that Cm5 was sufficient to inhibit growth of non-transformed *Leuc. mesenteroides* and *L. lactis*, and thus used as the selective concentration for these strains. Colonies were screened using the same PCR protocol as described for *E. coli*. All plasmids used in this study are listed in Table 2.

**Table 2:** Plasmids used in this study

Plasmid	Insert	Promoter (type)	Marker	Reference/source
pNZ44	–	P44 (constitutive)	CmR	McGrath et al.^92^
pNZSB1:*sacB_1*	*sacB_1*	PSB1 (inducible)	CmR	This study
pNZ44:*sacB_1*	*sacB_1*	P44 (constitutive)	CmR	This study

### Effect of sugar type and aeration conditions on *Leuc. mesenteroides* viability

Aeration conditions were defined based on the surface-to-volume ratio of the culture vessels. Low-aeration (LA) conditions were established by incubating 15 mL cultures in 15 mL conical Falcon tubes, minimizing headspace and reducing passive oxygen diffusion. High-aeration (HA) conditions were achieved by incubating the same culture volume (15 mL) in 100 mL Erlenmeyer flasks, providing a larger surface area for oxygen exchange. All cultures were incubated statically without shaking.

To validate the difference in oxygen availability, dissolved oxygen (DO) levels were measured using a portable DO meter probe. Measurements were taken on test vessels in duplicates after 6 h of incubation at 8 °C prior to inoculation, confirming low- and high-aeration flasks contained 5.3±0.2 mg/L and 10.9±0.3 mg/L DO, respectively.

*Leuc. mesenteroides* KS273 and KS276 were cultured at 8 °C in M17 broth supplemented with 1.5% sucrose, glucose, fructose, or no sugar under LA conditions for 120 h and samples were collected every 24 h for cell viability determination. In a second experiment, *Leuc. mesenteroides* KS273 and KS276 were incubated at 8 °C for 120 h in M17 broth supplemented with 1.5% sucrose or glucose, under both LA and HA conditions and samples were collected at the end of incubation for cell viability determination. In a third experiment, *Leuc. mesenteroides* KS276-E and KS276-SB1 were incubated at 8 °C for 120 h in M17 broth supplemented with 1.5% sucrose or glucose under LA conditions and samples were collected at the end of incubation for cell viability determination. In a fourth experiment, *L. lactis* MG1363-E and MG1363-SB1 were cultured under both LA and HA conditions in M17 broth supplemented with 1.5% sucrose at 30 °C for 48 h and samples were collected at the end of incubation for cell viability determination.

Bacterial cell viability was determined by serial dilution and plate counting. Samples were serially diluted 1:10 in sterile PBS, and aliquots were plated on GM17 agar. Plates were incubated at 30 °C for 48 h before colony-forming units (cfu) were enumerated.

### Zymogram

Zymogram analysis was conducted to detect sucrase activity in situ. 10% SDS-PAGE gels were prepared as described by Laemmli, using a Bio-Rad MiniProtean chamber (Bio-Rad Laboratories, Inc.). Whole cultures or PBS-washed cells pellets were mixed with SDS sample buffer without β-mercaptoethanol and treated at 70 °C for 5 min. Samples were centrifuged for 5 min at 15,000×*g*, supernatant was collected and 200 µg of total protein from samples was loaded into each gel well. Electrophoretic separation of proteins was performed, and following electrophoresis, the lane containing molecular mass reference marker was cut out and stained with Imperial Protein Stain (Thermo Fisher, Germany) according to the manufacturer’s instructions.

To renature the sucrases, lanes containing sample protein fractions were washed three times with 50 mM phosphate buffer (pH 6.5) containing 1% Tween 80 (Merck, Darmstadt, Germany), followed by an additional wash with Tween-free buffer. The renatured gel was then incubated overnight at 28 °C in 50 mM phosphate buffer (pH 6.5) containing 100 g/L sucrose. Following incubation, the gel was treated with 75% ethanol for 30 min for fixation, and subsequently incubated for 1 h in a solution containing 0.7% periodic acid and 5% acetic acid for protein oxidation.

After oxidation, the gels were washed three times for 20 min with a solution of 0.2% sodium metabisulfite and 5% acetic acid. Polymer production was detected by exposing the gels to Schiff’s reagent to stain the synthesized polymer.

### Statistical analyses

Statistical analyses were performed using GraphPad Prism v8 and Design Expert v11.1.2.0. For growth curves and viability assays, log_10_-transformed cfu/mL data were analysed using two-way repeated measures ANOVA, followed by Tukey’s multiple comparisons test to assess differences between groups at each timepoint. Where assumptions of normality or equal variance were not met non-parametric Kruskal–Wallis tests followed by Dunn’s post hoc test were applied. The level of statistical significance was set at *p* < 0.05. Experimental results are shown as mean ± standard deviation (SD) from at least three independent biological replicates. For RSM, a full factorial design was used to evaluate the effect and interaction of sugar concentration and temperature. Model quality was evaluated using regression coefficients (*R2*), lack-of-fit tests, and analysis of residuals.

## Results and discussion

### Sucrose and low temperature trigger EPS production

The production of slimy texture and EPS by four *Leuc. mesenteroides* strains (KS273, KS279, KS276, KS277) was evaluated across different carbon sources and incubation temperatures. Lower incubation temperatures consistently enhanced the ropy phenotype in the presence of sucrose, compared to fructose or glucose. This effect was most pronounced at 8 °C, where all strains exhibited visible thread formation, with the longest threads observed in 5% sucrose cultures. Strains KS273 and KS279 showed particularly strong responses under these conditions. At 25 °C, the ropy phenotype was generally milder and restricted to KS273 and KS279, and only in cultures supplemented with the highest sugar concentrations, with no substantial differences between sugar types. No ropy phenotype was detected in any strain at 37 °C under any condition.

**Fig. 1:**
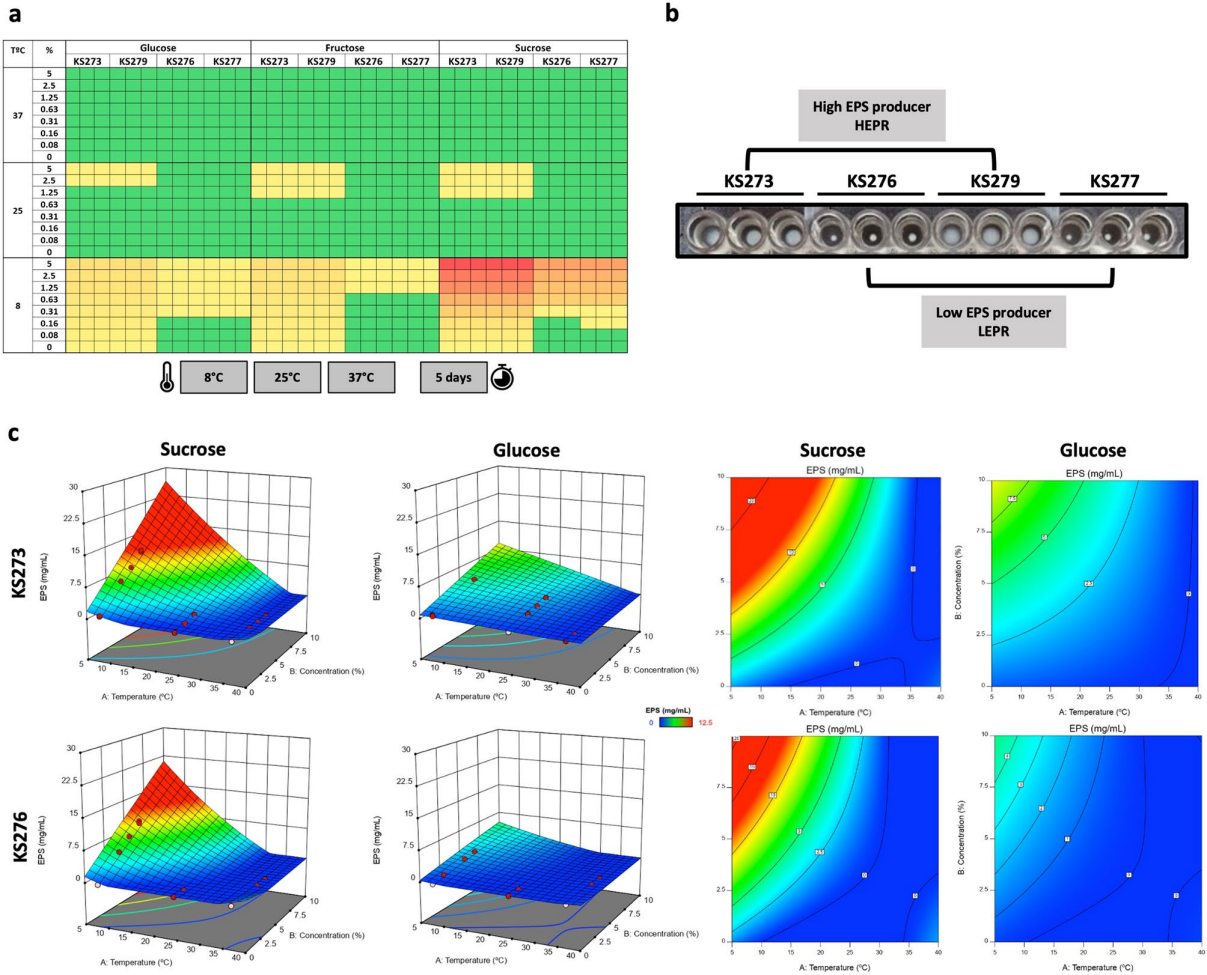
Effect of carbon sources and temperature on EPS production. **a** Heatmap representing the qualitative effect of temperature (8 °C, 25 °C, 37 °C) and carbon source (glucose, fructose, sucrose) on EPS production by four *Leuc. mesenteroides* strains (KS273, KS279, KS276, KS277). Higher EPS production is indicated by red/yellow coloration; **b** Visual comparison of high EPS producers (HEPRs, KS273, KS279) and low EPS producers (LEPRs, KS276, KS277) in round-bottom well microtiter plates after 5 days of incubation in 5% sucrose at 8 ºC; **c** Response Surface Methodology (RSM) 3D plots (left) and 2D contour plots (right) showing the quantitative effect of temperature and sugar (sucrose or glucose) concentrations on EPS production for *Leuc. mesenteroides* strains KS273 (top) and KS276 (bottom)

KS273 and KS279 were designated as HEPRs and KS276 and KS277 as LEPRs based on apparent visual and ropy phenotype differences (Fig. 1). HEPRs produced a large, thick and opaque pellet compared to LEPRs, which were clear and had less viscous appearance, indicative of lower EPS production.

### *Leuc. mesenteroides* KS273 produces more EPS than KS276 at low temperatures

Response surface modeling (Fig. 1c) revealed clear differences in EPS production depending on strain and sugar type. For *L. mesenteroides* KS273 and KS276, the model predicted maximum EPS yields of approximately 27 mg/mL and 20 mg/mL, respectively, at 5 °C and 10% sucrose—consistent with prior qualitative observations. In contrast, when glucose was used as the carbon source, predicted rEPS yields were substantially lower (~ 10 mg/mL for KS273 and ~ 8 mg/mL for KS276).

For both strains grown in sucrose, EPS production models were quadratic, with temperature appearing in the quadratic term (T2), indicating the relevance of this variable. In contrast, both models for glucose were linear. Notably, the four models exhibited a term showing the interaction between temperature and sugar concentration (TC), indicating these two factors synergistically drive maximal EPS production (Table 3).

**Table 3:** EPS production model equations for *Leuc. mesenteroides* strains KS273 and KS276

Strain	Sugar	EPS Model Equation (mg/mL)
KS273	Sucrose	EPS = 3.3358–0.3297T + 2.7234 C − 0.0799TC + 0.0068T^2^
KS273	Glucose	EPS = 0.9757–0.0294T + 0.9577 C − 0.0240TC
KS276	Sucrose	EPS = 3.4984–0.4494T + 2.3719 C − 0.0724TC + 0.0102T^2^
KS276	Glucose	EPS = 0.9294–0.1145T + 0.5822 C − 0.0172TC

T represents temperature (°C), and C represents sugar concentration (%)

The induction of sucrase-based HoPS biosynthesis by sucrose is widely reported^6,27^. Low temperature was demonstrated to be an important induction factor for both strains with both sugars. To the best of our knowledge, optimal EPS production at cold temperatures does not appear to be a typical phenotype across *Leuc. mesenteroides* strains described in the literature. However, production of dextran and dextransucrases has been reported in other *Leuconostoc* spp. such as *Leuc. lactis*, *Leuc. gasicomitatum*, *Leuc. gelidum*, and other LAB like *Weissella cibaria*^38–41^. To determine whether this phenotype is common among *Leuc*,* mesenteroides* strains, we screened a collection of 44 strains, including the KS strains, for ropy phenotype at 8 °C in M17 broth supplemented with glucose, fructose, or sucrose (Supplementary Table S1). Notably, only the KS strains exhibited a ropy phenotype under these conditions.

Most studies on *Leuc. mesenteroides* report that EPS production occurs between 20 and 30 °C, with some studies indicating no detectable EPS production below 15 °C^17,28,42–44^. However, these studies typically limited incubation to ≤ 36 h, which may have overlooked EPS production at low temperatures, where slower bacterial growth rates could require extended incubation times.

### *sacB_1* is a key genomic difference between HEPRs and LEPRs

A comparative genomic and pangenomic analysis of EPS biosynthetic clusters was conducted to elucidate the phenotypic differences between HEPRs and LEPRs.

**Fig. 2:**
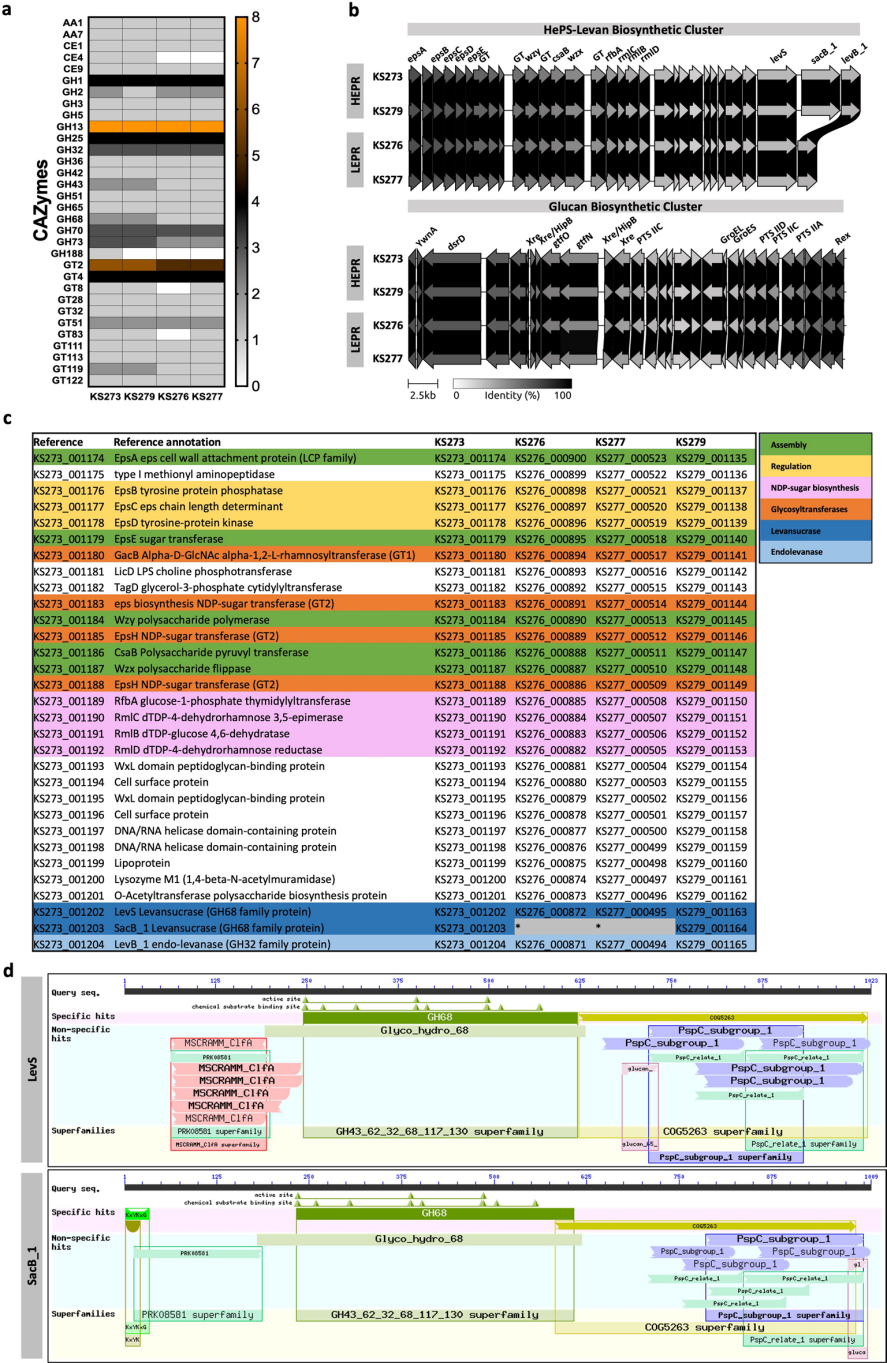
Genomic analysis of EPS biosynthetic clusters. **a** Heatmap of frequency of carbohydrate-active enzymes (CAZymes) from *Leuc. mesenteroides* high EPS producer strains (HEPRs: KS273, KS279) and low EPS producers (LEPRs: KS276, KS277); **b** Gene cluster organization of the proposed HePS-Levan (HLBC) and glucan (GBC) biosynthetic gene clusters in HEPRs and LEPRs. The gene clusters were visualized using Clinker on the CAGECAT platform, highlighting the levansucrase genes (*levS* and *sacB_1*) and glucansucrase genes (*dsrD*, *gtfN* and *gtfO*); **c** Annotation and presence/absence comparison of key proteins of the HLBC involved in HePS and levan biosynthesis pathways across the four *Leuc. mesenteroides* strains. Genes in the HePS operon were categorized into colour groups based on the putative or established functions of their products as in Zeidan et al. (2017); Poulsen, Derkx and Oregaard (2019)^10,90^: Modulation (yellow; phosphoregulatory module *epsBCD*), polysaccharide assembly (green; initiation *epsE*, polymerization *wzy*, export/flippase *wzx* and attachment *epsA*/*lytR*), GT (orange; glucosyltransferases) which assemble the repeating units, and non-housekeeping functions (pink) required for the synthesis of activated sugar precursors and modification of the sugar residues; **d** Conserved domains (CDs) of *levS* and *sacB_1* from the NCBI Conserved Domain Database^32^

A Carbohydrate Active Enzymes (CAZymes, Fig. 2a) analysis showed that HEPRs have a higher overall frequency of CAZymes compared to LEPRs, particularly in carbohydrate esterases (CE), glycoside hydrolases (GH) and glycosyltransferases (GT) families, which are critical for EPS biosynthesis. The CE4 (deacetylation of polysaccharides), GH43 and GH73 (associated with glycan hydrolysis and cell wall remodelling), GT2 and GT119 families (glycosyltransferase activity) were more abundant in HEPRs. This profile could point to a more efficient modification and maturation of EPS^45^polysaccharide degradation and restructuring, all processes that may enhance biofilm formation and EPS assembly^46,47^respectively. Interestingly, glucansucrase (GH70 family) genes were equally present in both groups. However, HEPR strains possess two genes of the GH68 family, while LEPRs possess only one. This family consists of frucosyltransferases (FTFs) that include levansucrase (EC 2.4.1.10), beta-fructofuranosidase (EC 3.2.1.26) and inulosucrase (EC 2.4.1.9).

In this regard, a more detailed analysis focused on GH68 and GH70 genes and their genomic context revealed significant insights into the gene clusters responsible for glucan, levan and HePS biosynthesis (Fig. 2b).

The Glucan Biosynthetic Cluster (GBC) was almost identical in the four strains indicating that the glucan production pathway is conserved across these strains. The overall genetic structure of GBC, with three putative glucansucrase (GH70 family) genes *dsrD*, *gtfN* and *gtfO*, and no accessory biosynthetic genes, is consistent with the production of glucose-based extracellular HoPS. Transcription factors (TFs) from the Rrf2, Xre, HipB and Rex families, chaperonins and PTS transporter genes were also present in the cluster (Fig. 2b). Among the glucansucrase genes, *dsrD* encodes the dextransucrase DsrD. This 170-kDa dextransucrase which primarily synthesizes soluble dextran with α-1,6 glycosidic linkages, typical for linear dextran structures. However, it may also incorporate a small fraction of branching linkages (e.g., α-1,3), depending on environmental conditions and the presence of certain acceptor molecules^48^. *gtfN* encodes a 1029-amino acid protein in KS273, KS279 and KS276, and a slightly truncated version of 994 amino acids in KS277. There is a putative promoter upstream of *gtfN* and *gtfO*. It may be that *gtfN-gtfO* was originally a single glucansucrase gene encoding for a 1512 amino acid protein, and a mutation produced an ORF shift and the consequent split into two smaller ORFs. The presence of a single promoter upstream of *gtfN*, the lack of both a KxYKxGKxW signal peptide and a complete GH70 domain in *gtfO*, and the lack of a complete glucan-binding repeat domain in *gtfN*, domains commonly found in glucansucrases, support this hypothesis^49^. A homology analysis of the two proteins fused as one against other glucansucrases showed a 98.8% identity with the dextransucrase DsrT from *Leuc. mesenteroides* NRRL B-512 F (GenBank: AB020020.1). Funane et al. (2000) reported that *dsrT* from this strain was truncated after the catalytic domain and produced an inactive enzyme by the deletion of five nucleotides. The insertion of five nucleotides at the putative deletion point in *dsrT* resulted in a 1512-amino acids enzyme with dextransucrase activity^50^. Our in-silico analysis showed that the insertion of five nucleotides in the deletion point of *gtfN* leads to the restoration of the complete ORF encoding a 1512-amino acid protein 99.8% identical to DsrT.

The genetic arrangement of GBC and the presence of *dsrD* and *dsrT* (truncated) genes indicate that GBC encodes sucrase-based machinery for the extracellular synthesis of dextran^6^.

*HePS-Levan biosynthetic cluster (HLBC)* The analysis of the genomic context of GH68 genes revealed a highly conserved cluster across KS strains, linked to the biosynthesis of HePS and levan (Fig. 2b, c) which closely resemble the Wzy-dependent EPS gene cluster described in seven *Leuc. mesenteroides* strains (Ln1–Ln7)^9^. Core components of the Wzy-pathway were found, such as *epsABCDE*, *wzy* (polysaccharide polymerase), *wzx* (polysaccharide flippase), 4 glycosyltransferase genes (GT1 and GT2 families) and 4 NDP-sugar biosynthesis genes^10^. Notably, *Wzy* (KS273_001184) and *Wzx* (KS273_001187) had no sequence similarity to their homologues in Ln1-Ln7 strains. Our annotation resulted from structure-based homology using the database “UniprotKB with 3D structures (AlphaFold)”. Functional confirmation through gene knockout and complementation should be performed in future studies to verify the roles of these genes. Additionally, experiments should be conducted to determine whether these clusters in KS strains are dedicated to rEPS or cell-bound EPS production. However, the presence of specific genes such as CsaB (KS273_001186, Pyruvyl-transferase involved in peptidoglycan-associated polymer biosynthesis) suggests that the produced polysaccharide undergoes pyruvylation via a conserved mechanism for the anchoring of proteins containing S-layer-homology (SLH) domain to the cell surface^51^suggesting the cell-bound type.

Three genes involved in levan metabolism were identified 9 kbp downstream of the HePS cluster (Fig. 2b). These include the levansucrase genes *levS* and *sacB_1* (GH68 family), responsible for levan synthesis^52^and the endo-levanase gene *levB_1* (GH32 family). Notably, *sacB*_*1* was present in HEPRs but not in LEPRs, suggesting that it could be at least partially responsible for the observed phenotypic differences. However, it is unlikely to be the sole contributor. Possibly additional regulatory elements and transport genes may also influence the overall EPS-overproduction phenotype in *Leuc. mesenteroides* strains.

The conserved domains (CDs) of *sacB_1* were analysed and compared to those of *levS*, which were characterized in previous studies on the *levS* gene from *Leuc. mesenteroides* NRRL B-512 F (GenBank: AAY19523.1, 99.9% identity) (Fig. 2d)^32^. The typical levansucrase structure comprises four regions: (i) a signal peptide (SP), (ii) a variable-length N-terminal stretch, (iii) a conserved catalytic core (~ 500 amino acids) shared among GH68 family members, and (iv) a C-terminal region, sometimes containing a cell wall binding domain (CBD)^6^. Some mosaic fructansucrases bear structural features of glucansucrases in the N- and C-terminal regions, such as C-terminal glucan-binding domains (GBDs) present in the levansucrases LevS, LevC, LevL, the inulosucrase IslA and others^52–55^. Both *levS* and *sacB_1* share the following CDs: a SP of 31 amino acids (MRKKLYKAGKLWVAGAAVAAAVVVAPNIVSA and MRKKLYKAGKIWVAGAATVAAISMGVSSVSA, respectively) with the KxYKxGKxW motif; an amidase domain in the N-terminal stretch; the GH68 catalytic core; a CBD (PspC subgroup 1) and a GBD (COG5263) in the C-terminal region. Notably, a ClfA adhesin clumping factor (MSCRAMM) domain was also identified in the N-terminal stretch of levS, which was not mentioned or characterised previously in levansucrases^6,52,53,56,57^.

The KxYKxGKxW motif and the CBD suggest that the levansucrases are secreted via an accessory Sec (aSec) or aSec-like mechanism^58^ and then displayed on the cell surface attached to the cell wall. The CBD composed of PspC subgroup 1 domains, with choline-binding ability, indicates protein attachment by non-covalent binding to choline molecules post-secretion^59,60^. Notably, DsrD and GtfN also contain these domains, suggesting a co-localization on the cell surface.

The ClfA domain in the N-terminal region of *levS* suggests an additional function involving adherence to host surfaces or substrates, potentially supporting biofilm formation. In *S. aureus*, the ClfA domain binds to fibrinogen, enabling stable bacterial attachment, crucial for robust biofilm formation^61^. This domain is present in levansucrases from *Leuc. mesenteroides*, such as LmLEVS (strain MTCC10508, GenBank: MH684490), LevS (strain NRRL B-512 F, GenBank: AAY19523.1), and L17 (strain Lm 17, GenBank: ALF07532.2), although its function remains uncharacterized^62^.

An amidase domain (PRK08581) is another feature not previously described in levansucrases studied in the literature, although it is present in LevS and Lm 17 from NRRL B-512 F and Lm 17 strains, respectively. Amidase domains are known to participate in the turnover and remodelling of peptidoglycan, allowing cell wall modifications in response to environmental changes^63^. Amidase activity may also contribute to peptidoglycan flexibility and stability, especially within biofilms where cell wall integrity is vital^64,65^.

GBD (YG-repeat, YG-box or COG5263) is a feature generally observed in glucansucrases, implicated in polymer chain growth and determining the structure of synthesized glucans^66,67^. In some GTFs, it is also essential for the retention of glucan synthesizing activity, as some become sucrose hydrolysing enzymes upon deletion of the YG box^68^. The inulosucrase IslA was found to be a natural chimeric fructosyltransferase formed by replacing the catalytic domain of alternansucrase (GH70 domain) with a fructosyltransferase (GH68 domain). The C-terminal domain may have provided an evolutionary advantage by anchoring inulosucrase to the cell surface while interacting with the catalytic core. It is proposed that this interaction stabilizes the enzyme, shifts its mechanism from hydrolysis to transglycosylation, and enhances both thermal stability and substrate specificity. Thus, chimeric constructions may become a strategy to stabilize and modulate biocatalysts based on FTF activity^54,55^.

Given the co-localization of glucansucrases and levansucrases on the cell surface and their GBD, we hypothesise that levansucrases may also participate in interactions with dextran within the EPS matrix. This could facilitate cross-linking of different polysaccharides like levan and dextran, promoting stable biofilm formation. Furthermore, levansucrases may serve as a cell anchor by binding dextran and leveraging its surface-adhesion properties^69^. Rozen et al. (2004) demonstrated that a FTF from *S. mutans* is adsorbed to cells grown in sucrose by interacting with high affinity with the cell-surrounding dextran, indicating that fructans and glucans are an integral part of the polysaccharide matrix of oral biofilms^70^. Experimental validation is required to confirm these hypotheses on *Leuc. mesenteroides* strains.

A pangenomic analysis was performed to compare the HLBC and GBC of *Leuc. mesenteroides* strain KS273 with other strains of *Leuc. mesenteroides* available in databases (Supplementary Fig. S1). The GBC showed a remarkable conservation across strains. This indicates that the cluster potentially contributes to the ecological fitness of these strains by enabling them to utilize sucrose efficiently or by playing a role in biofilm formation, protection under environmental stress and providing antimicrobial defences^71–73^.

Regarding HLBC, *levS and levB_1* were conserved in almost all clusters, while *sacB_1* was present in 42% of clusters. Interestingly, in clusters where *sacB*_*1* was present, these were notably incomplete compared to the HLBC of KS273/KS279, missing 7–11 genes related to the Wzy-HePS pathway, such as *epsE* (polysaccharide synthesis initiation sugar transferase), *wzy* (polysaccharide polymerase), *wzx* (polysaccharide export/flippase), glycosyltransferases and teichoic acid biosynthesis genes. Notably, *sacB_1* was absent in the only two clusters that were fully complete (*Leuc. mesenteroides* NCTC10817), suggesting a potential trade-off between the completeness of the HLBC and the presence of *sacB_1*. This may indicate that *sacB_1*-containing clusters undergo a loss of several genes associated with alternative or specialized EPS production mechanisms in these strains.

A phylogenetic analysis of *sacB_1* showed it is present in *Leuc. mesenteroides* strains isolated from a variety of foods of vegetable origin, such as birch sap, kimchi (70% of strains), fermented olives, winter pickled vegetables and soybean paste (Supplementary Table S3). To the best of our knowledge, KS273 and KS279 are the only meat-borne strains bearing SacB_1 reported to date. Sucrose and raffinose are natural inducers of gene expression as well as substrates for levansucrases^7^. In this light, the conservation of SacB_1 in these strains could be an evolutionary trait tied to the availability of sucrose and/or raffinose in these sources with different nutritional compositions but having one or two sugars in common. The sucrose concentration in the sausages where KS strains were sourced ranged from 1 to 1.5%. Birch sap contains approximately 1–2% sucrose as its dominant sugar, forming a readily available substrate for saccharolytic enzymes. Kimchi and Chinese winter pickled vegetables, primarily composed of cabbage, have lower sucrose levels, typically less than 0.5%. Still, these cruciferous vegetables are known for their high raffinose content, although no reports on concentrations were found. Additionally, sucrose is often added at 2–5% during fermentation to enhance microbial activity. Olives are low in sucrose, generally less than 0.1%, but sucrose is commonly introduced during brining, creating a favourable environment for microbial metabolism. On the other hand, soybean paste (doenjang) is rich in raffinose, and related oligosaccharides, which account for approximately 1–3% of its composition, providing an ideal substrate for SacB_1 activity^74–77^. The widespread occurrence of sucrose (and/or raffinose) in plant-based substrates and industrial meat sausages likely drove the selective pressure for enzymes that could efficiently leverage these carbohydrates. Levan production may offer further adaptive benefits by enhancing microbial resilience in dynamic fermentation ecosystems.

### Cold stress induces a metabolic switch towards Levan production

To elucidate why rEPS production is increased as the temperature lowers, why KS273 produces more rEPS than KS276, we examined the gene expression profiles of these two strains under sucrose (S), glucose (G), no sugar (NS) and two temperatures (25 and 8 ºC), with a particular focus on EPS-related genes.

**Fig. 3:**
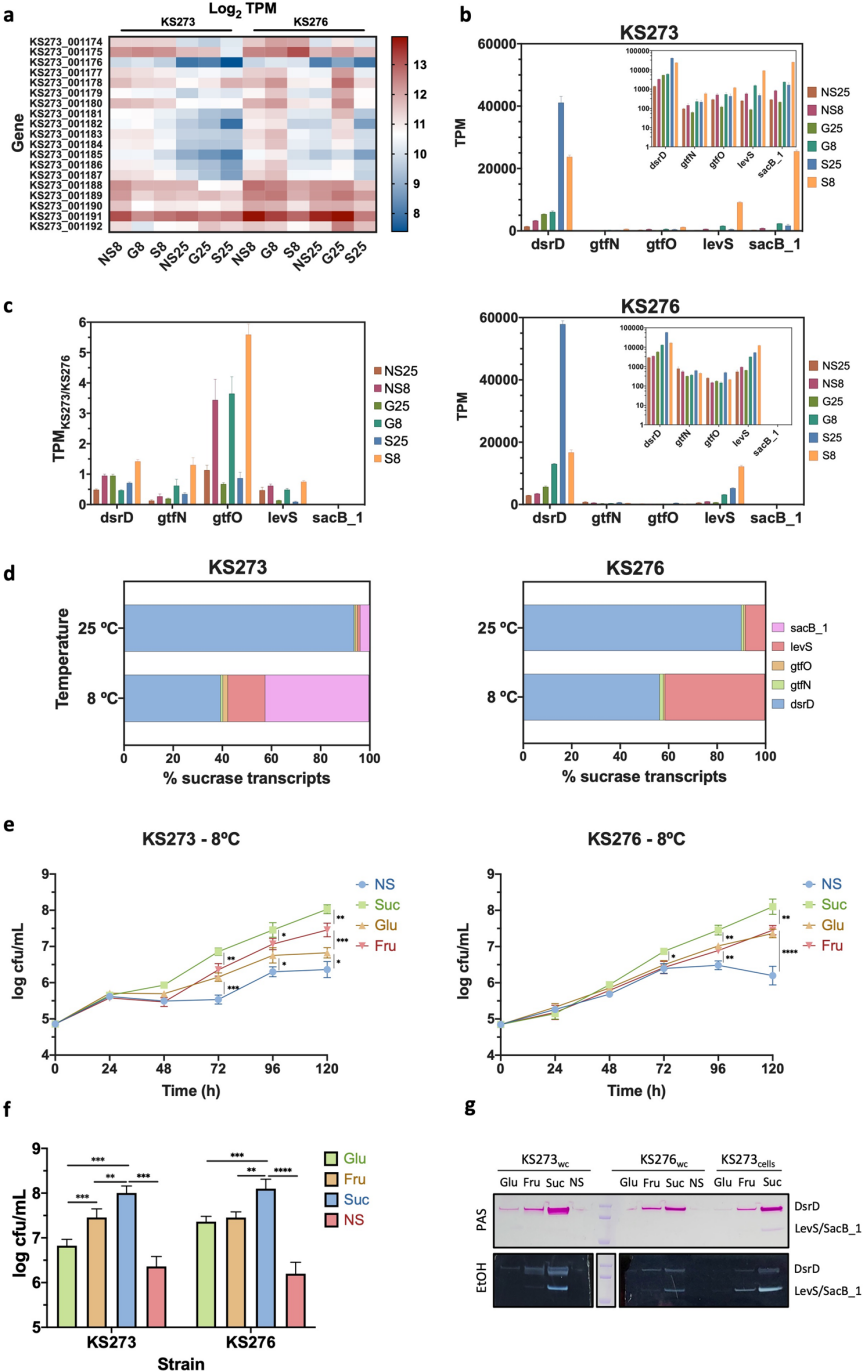
*Gene expression and viability analysis of Leuc. mesenteroides KS273 and KS276 under different carbon sources and temperatures.* **a** Heatmap of gene expression (Log2 TPM) of Wzy-dependent HePS biosynthesis pathway in KS273 and KS276; **b** Expression of sucrases genes (glucansucrases *dsrD*,* gtfN and gtfO* and levansucrases *levS*,* sacB_1*) in KS273 (top) and KS276 (bottom) cells grown in M17 with 1.5% glucose (G), sucrose (S) or no sugar (NS) at 25 or 8 ºC for 72 h, represented as transcripts per million (TPM); **c** ratio of KS273 and KS276 GTs TPM in each condition; **d** GT gene enrichment expressed as % GT transcripts in KS273 (left) and KS276 (right) at 25 ºC and 8 ºC in M17 with 1.5% sucrose; **e** Growth curve, **f** Final cell viability and **g** zymogram of GTs from KS273 (left) and KS276 (right) cultures grown in M17 with 1.5% glucose (Glu), sucrose (Suc), fructose (Fru) or no sugar (NS) at 8 ºC for 120 h. Zymogram displaying polymers synthesized in situ by DsrD (173 kDa), LevS (111 kDa) and SacB_1 (115 kDa) revealed by Periodic acid-Schiff (PAS) gel staining and ethanol polysaccharide precipitation. The latter is a composite image from two parts of the gel separated by the Commassie Blue-stained Protein Standard (10–200 kDa, New England Biolabs). Full-length gels are displayed in Supplementary Fig. S2. Data are presented as mean ± SD, *n* = 3. For growth curves and survival plot, two-way repeated measures ANOVA and Tukey’s post-hoc test was carried out: ****p < 0.0001, ***p < 0.001, **p < 0.01, *p < 0.05

The Wzy-dependent HePS cluster within HLBC (Fig. 3a) showed increased expression at 8 ºC compared to 25 ºC in both strains, particularly in NS and G conditions. Notably, G25 also induced high expression in KS276. In addition, KS276 showed higher cluster transcription levels than KS273, suggesting it may produce more HePS than the latter. Overall, HePS production in both strains was induced at low temperatures in the absence of sucrose. HePS often plays a role in adhesion and biofilm formation, increasing in response to environmental and nutritional stresses like non-temperatures, acids, and low or alternative carbon source availability, as well as during infective processes in the case of pathogens. This may enhance bacterial survival by providing a protective barrier against desiccation, oxidative or osmotic stress, or immune recognition^1^. In this light, KS276 could be better adapted to environmental responses by a HePS production mechanism than KS273.

We also studied the expression of levansucrase and dextransucrase genes in both strains (Fig. 3b). We found that nearly all levansucrase and dextransucrase genes exhibited significantly higher expression at cold exposure in both strains. Sucrases transcription levels in this condition were even higher than in the HePS biosynthesis cluster. This points to an adaptive response favouring levan and dextran HoPS biosynthesis in this condition, likely as the primary protective mechanism against cold stress. In contrast to levansucrases, *dsrD* exhibited significantly higher expression at 25 °C than at 8 °C under sucrose conditions, suggesting a preferential role for dextran synthesis at warmer temperatures. Notably, the TPM values of *dsrD* at 25 ºC were approximately 10–100 times higher than those of the levansucrases, indicating a metabolic preference for dextran production rather than levan production in sugar-rich environments at warmer conditions.

In contrast to HePS production, sucrose is the most effective carbon source for inducing sucrase gene expression in both strains, followed by glucose and then no sugar. These results indicate that the rEPS produced from sucrose at low temperatures are predominantly levan and dextran, and the lower rEPS amounts found in glucose are predominantly HePS. However, rEPS characterization is required to confirm this hypothesis.

This substrate preference is consistent with other studies of *Leuconostoc* species, where sucrose has been shown to be a potent inducer of glucansucrase and levansucrase activity, leading to increased EPS production^8,27,78^. However, significant gene expression and sucrase activity can be induced with alternative carbon sources in *Leuc. mesenteroides*, such as raffinose, fructose, maltose or xylose^7,8,27,53,79^.

In addition to EPS-related genes, transcriptomic analysis after 72 h at 8 °C revealed upregulation of several genes typically associated with bacterial cold stress responses. These included cold shock proteins (Csp family), ribosome-associated factor A (*rbfA*), RNA helicases, and the molecular chaperone *dnaJ*. The induction of these genes supports the activation of known cold-adaptive mechanisms, confirming that 8 °C imposes a physiological stress on *Leuc. mesenteroides* under the tested conditions.

Under cold stress, bacterial transcription and translation undergo significant modifications to adapt to the lower temperatures. Upon exposure to cold, overall gene expression slows down as mRNA secondary structures stabilize, hampering transcription and translation initiation^80^. A higher transcription level of crucial stress-relief genes is usually associated with adapting microorganisms to stress conditions^81,82^. When analysing the KS273/KS276 TPM ratio for each gene (Fig. 3c), we observed that the values at 8 ºC were 2 to 6.5 times higher than those at 25 ºC under the same sugar conditions. This indicates that KS273 exhibits a better adaptive response to cold stress than KS276 in terms of sucrase expression. However, KS276 seems to have a better adaptive response to cold stress regarding HePS production, as explained previously (Fig. 3a).

Since sucrose was the most triggering carbon source, we compared the contribution of each sucrase gene transcription to the total sucrases transcription at each temperature in the presence of this sugar (Fig. 3d). At 25 °C, the sucrase expression profile was dominated by *dsrD* (blue segment), constituting 90–93% of the total sucrase transcripts while *levS* (red segment) and *sacB_1* (pink segment) contribute minimally to it in both strains, indicating that their expression is significantly repressed or basal. In contrast, at 8 °C, the expression profile changes drastically; *dsrD* shows a marked decrease to 39% and 56% for KS273 and KS276, respectively. Meanwhile, the levansucrase genes are up 57% in KS273 and 41% in KS276. This shift indicates a temperature-sensitive regulatory mechanism inducing a metabolic switch towards levansucrase biosynthesis, where its expression is enhanced in colder environments. This is likely to increase the production of levan which may provide adaptive benefits under cold stress.

Although we observed strong upregulation of *sacB_1* under cold stress, the molecular mechanism underlying its temperature-sensitive expression remains unknown. It is possible that *sacB_1* is regulated by temperature-responsive elements, such as cold-shock proteins or specific transcriptional regulators that enhance expression under low temperatures. Alternatively, the gene may be controlled by a cold-inducible promoter or by changes in DNA supercoiling affecting promoter accessibility. Future work including promoter mapping, mutational analyses, and identification of regulatory proteins will be necessary to elucidate the mechanisms that govern *sacB_1* expression under cold stress.

We studied the impact of sucrose, fructose, glucose or no sugar on the cell viability of each strain over time under cold stress at 8 ºC and LA conditions (Fig. 3e, f). For both strains, growth increased consistently over time with all carbon source conditions, but it was markedly enhanced in the presence of sucrose leading to the highest bacterial counts, followed by fructose and then glucose. The no-sugar (NS) control condition showed significantly lower growth, indicating that both strains rely heavily on an external carbon source for optimal growth under cold stress. Notably, KS276 reached maximum growth in the NS control condition at 72 h, with a decline in viability observed after 96 h, while KS273 maintained stable viability over the same period. Another key difference between the two strains is that the growth curves of KS273 in the different carbon sources began to markedly diverge from 72 h onwards, with a viability pattern being sucrose > fructose > glucose > NS until the end of incubation (*p* < 0.01). A similar pattern was clearly observed for KS276 only at the end of incubation, with the exception that no significant difference was found between growth in fructose and glucose.

A zymogram (Fig. 3g) carried out on samples from the end of this experiment showed that the dextransucrase DsrD and the two levansucrases are produced in different amounts in each sugar condition, with sucrose > fructose > glucose > no sugar, which agrees with the gene expression results and the growth curve patterns described previously. These observations are consistent with an interpretation that the increase in both DsrD and levansucrases expression is responsible for better cell fitness or adaptation to cold stress leading to higher survival.

Dextransucrases have been demonstrated to exhibit increased expression and activity in LAB under low-temperature conditions, indicating their role in environmental adaptation. For instance, in *Leuc. lactis* AV1n, dextran production and dextransucrase gene (*dsrLL*) expression were significantly higher at 20 °C compared to 37 °C, particularly in the presence of sucrose^39,40^. *Leuc. gelidum* and *Leuc. gasicomitatum* spoilage isolates have been shown to produce dextran-based slime in herring conserve after two weeks at 0–6 °C^41^highlighting the dextransucrase’s role in cold adaptation and biofilm formation in *Leuconostoc* spp. Similarly, *W. cibaria* displayed maximum dextransucrase activity and dextran yields at 15 °C during cold-shift sourdough fermentation in the absence of sucrose, further linking low temperatures to increased rEPS production^38^. Bacterial fructans have also been linked to stress resilience, aiding survival under nutrient limitation and membrane stress, although no reports on increased levan or fructan production under cold exposure were found for LAB^11,12^.

### Sucrose induced metabolism (SIM) and SacB_1-derived Levan protect *Leuc. mesenteroides* from oxidative stress

**Fig. 4:**
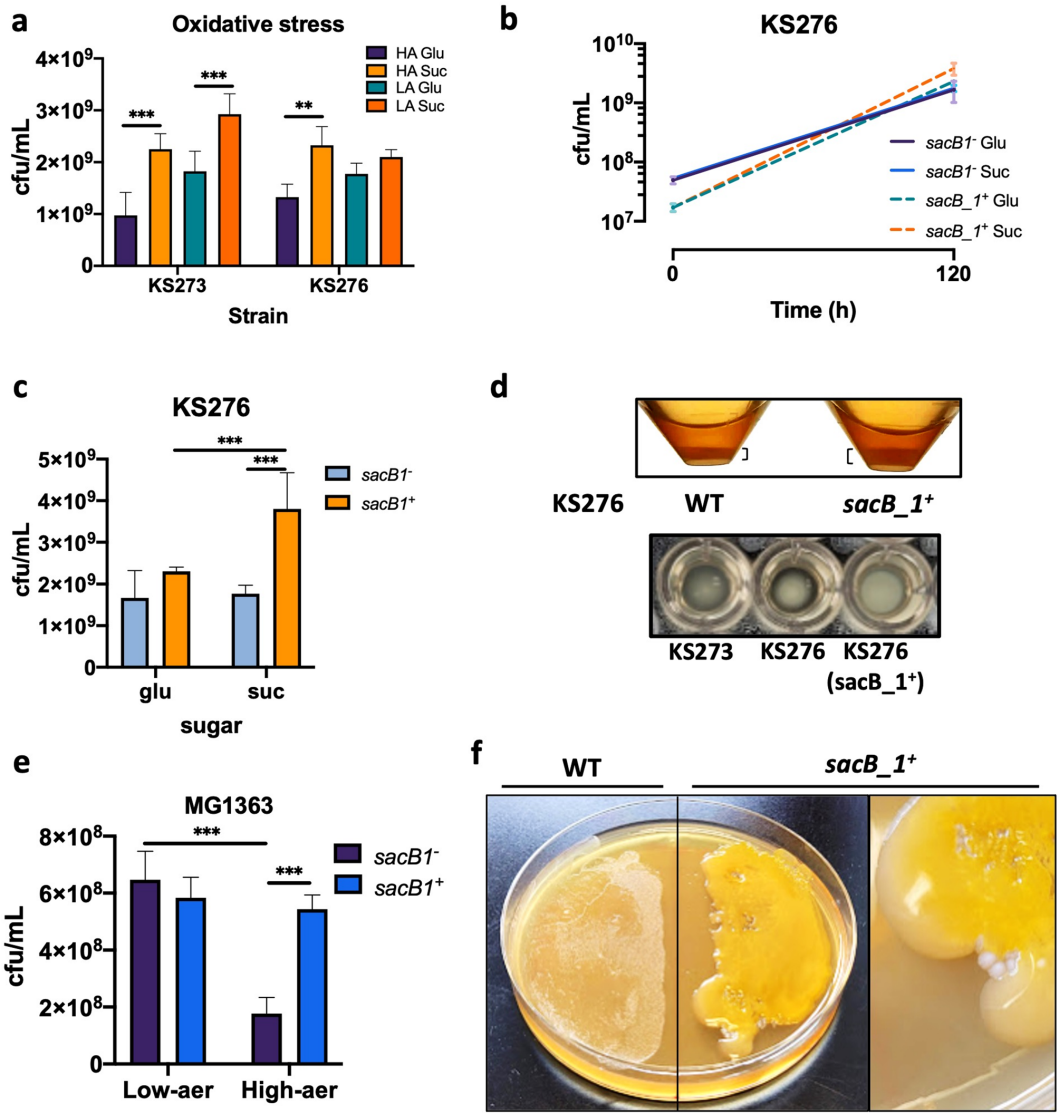
Protective effect of sucrose-induced metabolism (SIM) and levan. **a** Viability of *Leuc. mesenteroides* KS273 and KS276 after growing in different levels of oxidative stress: high aeration, (high surface: volume ratio, HA) or low aeration (low surface: volume ratio, LA) in the presence of glucose (Glu) or sucrose (Suc); **b** growth trend and **c** final viability of KS276-E (*sacB_1*^−^) and KS276-SB1 (*sacB_1*^+^) at 8 ºC under LA in presence of Glu or Suc. **d** Cell pellets of KS276 (WT) and KS276-SB1 (*sacB_1*^+^) after growth (120 h) in the presence of Suc under HA in falcon tubes (top) or round-bottom 96-well plate (bottom). **e** Viability of *L. lactis* MG1363 (*sacB_1*^−^) and MG1363-SB1 (*sacB_1*^+^) after growing for 24 h at 25 ºC in HA or LA in the presence of Suc. f) *L. lactis* MG1363 (WT) and MG1363-SB1 (*sacB_1*^+^) colonies after growing for 48 h at 25 ºC on M17 agar plates supplemented with 1.5% sucrose. Data are presented as mean ± SD, *n* = 3. Kruskal–Wallis test and Dunn’s post-hoc test: ***p < 0.001, **p < 0.01, *p < 0.05

Given that some fructans, glucans and mixtures of both possess antioxidant properties^14–18,26,83,84^ and oxygen concentration increases in aqueous systems as temperature drops, we investigated whether the improved *Leuc. mesenteroides* survival in the presence of sucrose could be due, at least in part, to the protection against oxidative stress by rEPS produced under sucrose-induced metabolism (SIM). We assessed the viability of KS273 and KS276 under LA and HA conditions after growing in M17 broth supplemented with glucose or sucrose at 8 ºC (Fig. 4a). The overall trend showed that aeration affects the viability of both strains, having lower viability under HA. SIM improved viability under both aeration conditions, although the protective effect was particularly evident for KS273. This effect could be attributed to the extended levansucrase capacity of KS273 due to the presence of *sacB_1.* To test this hypothesis, KS276 was transformed with the plasmid pNZSB1:*sacB_1*, containing *sacB_1* under the control of its putative promoter PSB1, or with the empty plasmid pNZSB1 as control. The growth and viability of the control (KS276-E, *sacB_1*^*−*^) and test strain (KS276-SB1, *sacB_1*^*+*^) were evaluated under LA at 8 ºC in glucose or sucrose (Fig. 4b and c). KS276-SB1 grew consistently better than KS276-E in both sugars, particularly in sucrose, in which the final viability of KS276-SB1 doubled that of the negative control (*p* < 0.001), even though the initial cell concentration of the latter was 0.5 log higher. A similar trend was observed for glucose, although no statistical significance was found. Notably, KS276-SB1 produced thicker cell pellets and larger and slimier colonies than the WT strain when growing in sucrose-containing M17 broth or agar plates at 8 ºC (Fig. 4d).

Attempts were made to generate a Δ*sacB_1* knockout mutant in strain KS273 for functional characterisation and complementation studies. However, repeated efforts to introduce the required plasmids into this wild-type strain were unsuccessful, despite eventual successful transformations in KS276 using the same vectors. This suggests that strain-specific differences, possibly related to restriction–modification systems, cell envelope properties, or natural competence, may limit plasmid uptake in KS273. We acknowledge this limitation and are currently exploring alternative strategies to overcome these barriers in future work.

To evaluate the isolated protective effect of SacB_1 against oxidative stress, *L. lactis* MG1363 was also engineered to express the *sacB_1* gene (MG1363-SB1, *sacB_1*^+^) under the control of the strong constitutive lactococcal promoter P44. The generation of reactive oxygen species (ROS) at high oxygen levels can overwhelm the antioxidant defence systems of *L. lactis*, leading to cellular damage and decreased viability^85,86^. The cell viability of MG1363-SB1 and MG1363-E strain, carrying the empty plasmid pNZ44, were assessed under HA and LA conditions in presence of sucrose (Fig. 4e). Under LA, both strains exhibited similar viability. In contrast, the engineered MG1363-SB1 strain showed enhanced cell viability compared to MG1363-E under HA conditions, being approximately three times higher and reaching similar viability levels as under low oxygen conditions (LA).

This result indicates that the protective role of SacB_1 is primarily beneficial in environments with relatively high oxygen concentration, hence high oxidative stress, while its effect is less pronounced when oxygen concentration is relatively low. The increased viability observed in MG1363-SB1 compared to MG1363-E under HA conditions supports the hypothesis that levansucrase SacB_1 plays a role in protecting the cell from oxidative damage.

Furthermore, plating for colonies (Fig. 4f) revealed remarkable differences in colony morphology and growth characteristics between MG1363-SB1 and WT MG1363 growing in sucrose. The WT strain showed typical *L. lactis* colony morphology. In contrast, MG1363-SB1 displayed a dense yellow rEPS with mucoid characteristics completely covering the typical *L. lactis* colonies. This visual observation aligns with the quantitative results, where MG1363-SB1 is separated from the environment and likely protected from oxygen by the dense levan layer, probably decreasing cell stress and improving viability under high oxygen concentrations.

Although levans role in *Leuconostoc* biofilms has not been reported to the best of our knowledge, we found that the endolevanase gene *levB_1* in both strains was upregulated at 8 ºC (Log2FC 0.89–1.41), correlating with levansucrase expression, indicating that levan serves as a nutritional source for the cells. In contrast, KS strains do not possess dextranases (GH31, GH49, GH66 families), pointing that dextran does not fulfil a direct nutritional role in these strains but probably a role in biofilm formation and/or an indirect role as carbon and energy source in a complex microbiological ecosystem. On the sausage surface, microbial cohabitation could influence dextran utilization. For instance, *Lactobacillus* or *Streptococcus* species produce dextranases, which degrade dextran into oligosaccharides such as isomaltooligosaccharides or glucose, facilitating mutualistic interactions^87–89^. Furthermore, dextrans have been demonstrated to provide antioxidant protection as well^18,25^. Interestingly, a mixture of dextran and levan with high molecular weight produced by *Leuc. mesenteroides* DSA_F exhibited antioxidant activity and provided mild protection to DNA from oxidative damage^18^suggesting that the same phenomenon could be happening in KS strains at cold temperatures. However direct antioxidant activity of EPS produced by KS strains remains to be determined.

Altogether, these findings observed in *Leuc. mesenteroides* KS273, KS276, and *L. lactis* MG1363 support the hypothesis that the metabolic switch under SIM at cold exposure leading to high production of sucrases, levan and dextran helps mitigating oxidative stress and very likely other types of stressors. This points to an adaptive mechanism and evolutionary advantage conferred by levansucrases in *Leuc. mesenteroides* and reveals a potential strategy for mitigating food spoilage through targeted microbial management. We confirmed the protective properties of levan; however, additional experiments should be conducted to verify whether levan-dextran mixture further enhances protection and cell survival in these conditions and the corresponding mechanisms.

## Conclusions

This study provides new insights into the regulation of EPS production in *Leuc. mesenteroides* under cold stress conditions. We identified *sacB_1*, a previously uncharacterized levansucrase gene, which was uniquely present in high EPS-producing meat-derived strains. Our findings reveal that low temperatures and sucrose availability induce a metabolic shift favouring levan production, linked to improved bacterial survival under oxidative stress. The temperature-sensitive regulation of *sacB_1*, *levS* and *dsrD* expression, together with traditional sucrase pathways, highlights an adaptive strategy for persistence in cold, nutrient-rich environments such as refrigerated meat products.

Importantly, this is the first study to associate the presence of *sacB_1* and *levS* with cold-responsive EPS biosynthesis in *Leuc. mesenteroides*, expanding the current understanding of microbial adaptation mechanisms. The identification of strain-specific EPS regulation has broader implications for controlling spoilage in food systems and for developing targeted strategies to harness EPS properties in biotechnology and health-related applications. These findings reveal new insights into sucrase diversity as an adaptive trait in LAB, with direct implications for microbial survival strategies, food spoilage control, and biotechnological applications.

## Electronic supplementary material

Below is the link to the electronic supplementary material.

Supplementary Material 1

## Data Availability

Assembled genomes were deposited to GenBank and raw RNA-seq data were deposited to the NCBI Sequence Read Archive (SRA) under BioProject accession number PRJNA1226170. Additional datasets used and/or analysed during the current study are available from the corresponding author on reasonable request.
