# Supplementary material for: Cold induced expression of a novel levansucrase gene sacB1 enhances exopolysaccharide production and stress resilience in Leuconostoc mesenteroides
Source: Sci Rep. 2025 Jul 2;15:22980. doi: 10.1038/s41598-025-04141-x (PMC12215647; doi:10.1038/s41598-025-04141-x)
Supplement: Supplementary file 1 — Supplementary Material 1 [file 41598_2025_4141_MOESM1_ESM.pdf]

Supplementary information

Cold induced expression of a novel levansucrase gene *sacB1* enhances exopolysaccharide production and stress resilience in *Leuconostoc mesenteroides*

Miguel Fernandez de Ullivarri, Colin Buttimer, Janneke Wijman, Eelco Heintz, Paul Ross, Matthew McCusker, Colin Hill*

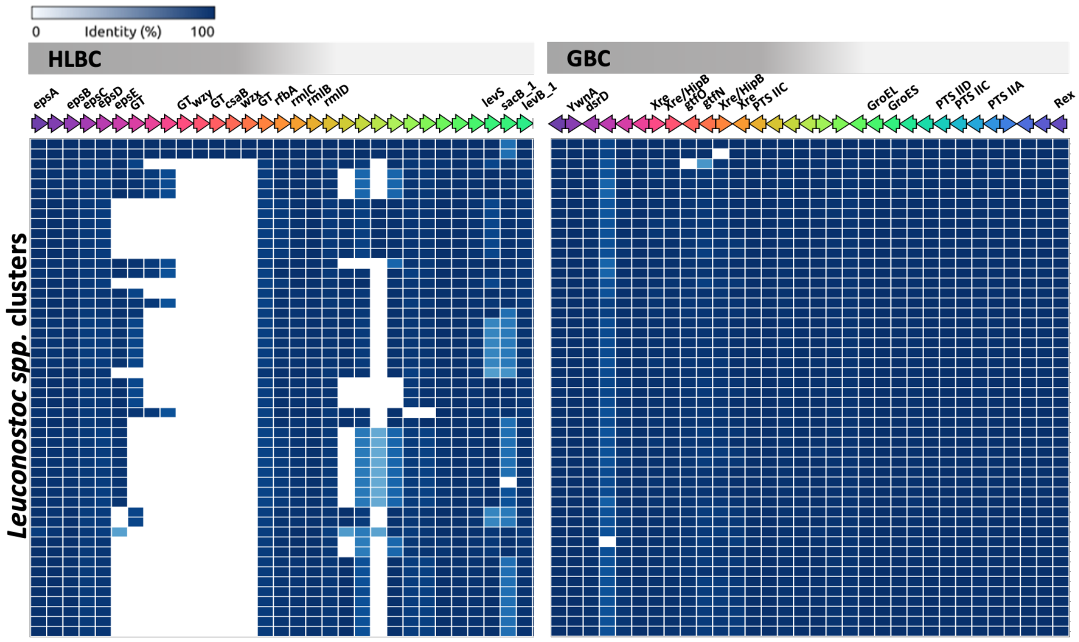

Supplementary Figure S1. Comparative pangenomic analysis of the HLBC and GBC from *Leuc. mesenteroides* KS273 with other *Leuconostoc spp.* strains from NCBI Database. Homologous gene clusters were identified using cblaster tool in CAGECAT platform with a 70% sequence identity threshold.

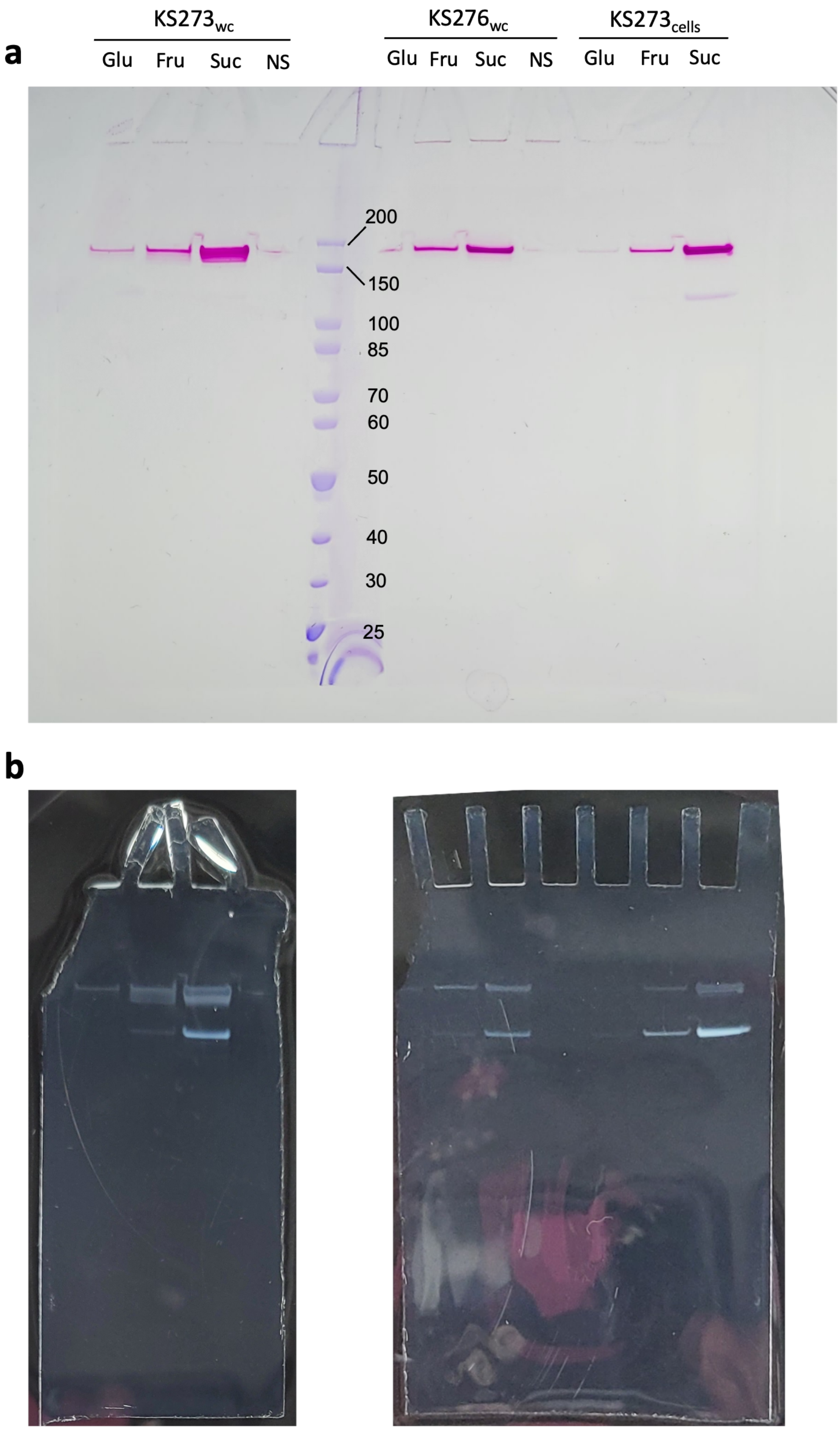

Supplementary Figure S2. Full length zymograms displaying polymers synthesized in situ by DsrD (173 kDa), LevS (111 kDa) and SacB_1 (115 kDa) revealed by a) Periodic acid-Schiff (PAS) gel staining and b) ethanol polysaccharide precipitation.

Supplementary Table S1. Ropy test analysis on a panel of *Leuc. mesenteroides* strains grown in different carbon sources at 8 ºC for 120 h.

Species	Strain	Description	Reference/Source	Ropy Test 8 ºC		
				Glu	Fru	Suc
Leuc. mesenteroides subsp. dextranicum	KS273	Food borne; Low temp. strong slime former	This study	2+	2+	4+
Leuc. mesenteroides subsp. dextranicum	KS276	Food borne; Low temp. weak slime former	This study	1+	1+	3+
Leuc. mesenteroides subsp. dextranicum	KS277	Food borne; Low temp. weak slime former	This study	1+	1+	3+
Leuc. mesenteroides subsp. dextranicum	KS279	Food borne; Low temp. strong slime former	This study	2+	2+	4+
Leuc. mesenteroides	KS12	Food borne strains	This study	-	-	-
Leuc. mesenteroides	KS50	Food borne strains	This study	-	-	-
Leuc. mesenteroides	KS52	Food borne strains	This study	-	-	-
Leuc. mesenteroides	P1A1	Food borne strains	This study	-	-	-
Leuc. mesenteroides	CURRENT2901	Food borne strains	This study	-	-	-
Leuc. mesenteroides	CURRENT2902	Food borne strains	This study	-	-	-
Leuc. mesenteroides	CURRENT2903	Food borne strains	This study	-	-	-
Leuc. mesenteroides	KS580-1	Food borne strains	This study	-	-	-
Leuc. mesenteroides	M120202	Food borne strains	This study	-	-	-
Leuc. mesenteroides	P2-1714	Food borne strains	This study	-	-	-
Leuc. mesenteroides	P3-1715	Food borne strains	This study	-	-	-
Leuc. mesenteroides	P3-1711	Food borne strains	This study	-	-	-
Leuc. mesenteroides	CC1-0601	Food borne strains	This study	-	-	-
Leuc. mesenteroides	B103003	Food borne strains	This study	-	-	-
Leuc. mesenteroides	B103005	Food borne strains	This study	-	-	-
Leuc. mesenteroides	PB1602	Food borne strains	This study	-	-	-
Leuc. mesenteroides	PB-A1	Food borne strains	This study	-	-	-
Leuc. mesenteroides	PC-D1	Food borne strains	This study	-	-	-
Leuc. mesenteroides	VBEM2	Food borne strains	This study	-	-	-
Leuc. mesenteroides	VBET2	Food borne strains	This study	-	-	-
Leuc. mesenteroides	VBPEM1	Food borne strains	This study	-	-	-
Leuc. mesenteroides	VBPEM2	Food borne strains	This study	-	-	-
Leuc. mesenteroides	VBPET2	Food borne strains	This study	-	-	-
Leuc. mesenteroides	VSEM2	Food borne strains	This study	-	-	-
Leuc. mesenteroides	VSET1	Food borne strains	This study	-	-	-
Leuc. mesenteroides	VSET2	Food borne strains	This study	-	-	-
Leuc. mesenteroides	VSET3	Food borne strains	This study	-	-	-
Leuc. mesenteroides	VSET6	Food borne strains	This study	-	-	-
Leuc. mesenteroides	VSPET1	Food borne strains	This study	-	-	-

Leuc. mesenteroides	VCEM3	Food borne strains	This study	-	-	-
Leuc. mesenteroides	VCET2	Food borne strains	This study	-	-	-
Leuc. mesenteroides	CRMFB(A) 1M	Food borne strains	This study	-	-	-
Leuc. mesenteroides	CRMFB(A) 2M	Food borne strains	This study	-	-	-
Leuc. mesenteroides	CRMFB(B) 1M	Food borne strains	This study	-	-	-
Leuc. mesenteroides	APC 2294	Food borne strains	This study	-	-	-
Leuc. mesenteroides	APC 2143	Food borne strains	This study	-	-	-
Leuc. mesenteroides	APC 2137	Food borne strains	This study	-	-	-
Leuc. mesenteroides	5.4(APC)	Food borne strains	This study	-	-	-
Leuc. mesenteroides subsp. dextranicum	DSM 20240	Type strain; EPS producer	DSMZ culture collection	-	-	-
Leuc. mesenteroides subsp. mesenteroides	DSM 20343	Type strain; EPS producer	DSMZ culture collection	-	-	-

Supplementary Table S2. Primers used in this study

Primer	Sequence	Template	Tann [ºC]	Product	Purpose	Amplicon size
NcoI-Lev2-for	cgg**ccatgg**aggaggaaaaATGAGAAAGAAGTTATACAAGGCG					
XhoI-Lev2-rev	gcc**ctcgag**CTGCTTTAAAATCAGGATTGCTAT	KS273 DNA	61	*sacB_1*	Cloning into pNZ44	3116
NcoI-Prom_Lev2-for	cgg**ccatgg**CTGTAATCTATGGCTACACTCTG					
XhoI-Lev2-rev	gcc**ctcgag**CTGCTTTAAAATCAGGATTGCTAT	KS273 DNA	61	*P_sacB_1*	Cloning *P_sacB_1* into pNZ	3430
XhoI-pNZ-for	gcc**ctcgag**TGCATATTTTCGGCAATCTTCTC					
NcoI-pNZ-rev	cgg**ccatgg**TCCCGTCTCCTGGATGG	pNZ44	61	pNZ vector (promoterless)		2517
pNZ44-for	CTAATGTCACTAACCTGCCCCGTTAG					
pNZ44-rev	GGCTATCAATCAAAGCAACACGTG	pNZ44-based vectors	61	Region of MCS	Selection of positive clones	variable

Sequence in bold indicates restriction site. Underline sequence indicates the Shine-Dalgarno sequence.

Supplementary Table S3. Comparative phylogenetic analysis of the *sacB_1* gene occurrence in other *Leuc. mesenteroides* strains in NCBI database.

Strain	Query Cover	% Ident	Accession	Source
Leuc. mesenteroides Lm10	100%	99.4	CP147495.1	Birch sap
Leuc. mesenteroides SRCM217106	100%	99.27	CP128496.1	Kimchi
Leuc. mesenteroides SRCM103356	100%	99.27	CP035139.1	Kimchi
Leuc. mesenteroides CBA7131	100%	98.88	CP021966.1	Kimchi
Leuc. mesenteroides subsp. mesenteroides ATCC 8293	100%	98.88	CP000414.1	Fermented olives

Leuc. mesenteroides FDAARGOS_1033	100%	98.88	CP065995.1	Clinical
Leuc. mesenteroides PL03	100%	98.79	CP103383.1	Kimchi
Leuc. mesenteroides SRCM103460	100%	98.79	CP035746.1	Kimchi
Leuc. mesenteroides MSL129	100%	98.79	CP128560.1	Kimchi
Leuc. mesenteroides subsp. mesenteroides CBA3650	100%	98.73	CP159465.1	Kimchi
Leuc. mesenteroides subsp. mesenteroides MSJK0064	100%	98.73	CP136668.1	Winter pickled vegetables
Leuc. mesenteroides WiKim0121	100%	98.37	CP098784.1	Kimchi
Leuc. mesenteroides WiKim32	100%	98.28	CP037752.1	Kimchi
Leuc. mesenteroides subsp. mesenteroides J18	97%	99.25	CP003101.3	Kimchi
Leuc. mesenteroides subsp. mesenteroides DRC0211	97%	99.25	CP013016.1	Kimchi
Leuc. mesenteroides SRCM102735	100%	98.09	CP028255.1	Soybean paste
Leuc. mesenteroides ML2	100%	98.09	CP143438.1	Fermented soybean
Leuc. mesenteroides WiKim33	100%	98.09	CP021491.1	Kimchi
Leuc. mesenteroides SRCM103453	100%	98.09	CP035271.1	Kimchi
